# Viral Membrane Fusion: A Dance Between Proteins and Lipids

**DOI:** 10.1146/annurev-virology-111821-093413

**Published:** 2023-09-29

**Authors:** Judith M. White, Amanda E. Ward, Laura Odongo, Lukas K. Tamm

**Affiliations:** 1 Department of Cell Biology, University of Virginia, Charlottesville, Virginia, USA; 2 Center for Membrane and Cell Physiology, University of Virginia, Charlottesville, Virginia, USA; 3 Department of Molecular Physiology and Biological Physics, University of Virginia, Charlottesville, Virginia, USA

**Keywords:** class I viral fusion proteins, class II viral fusion proteins, class III viral fusion proteins, conformational intermediates, trimers-of-hairpins, fusion energetics, lipid dynamics, fusion peptides, fusion loops, fusion restriction factors

## Abstract

There are at least 21 families of enveloped viruses that infect mammals, and many contain members of high concern for global human health. All enveloped viruses have a dedicated fusion protein or fusion complex that enacts the critical genome-releasing membrane fusion event that is essential before viral replication within the host cell interior can begin. Because all enveloped viruses enter cells by fusion, it behooves us to know how viral fusion proteins function. Viral fusion proteins are also major targets of neutralizing antibodies, and hence they serve as key vaccine immunogens. Here we review current concepts about viral membrane fusion proteins focusing on how they are triggered, structural intermediates between pre- and postfusion forms, and their interplay with the lipid bilayers they engage. We also discuss cellular and therapeutic interventions that thwart virus-cell membrane fusion.

## THE BASIC WHY, WHERE, AND HOW OF VIRAL MEMBRANE FUSION

**Figure 1:**
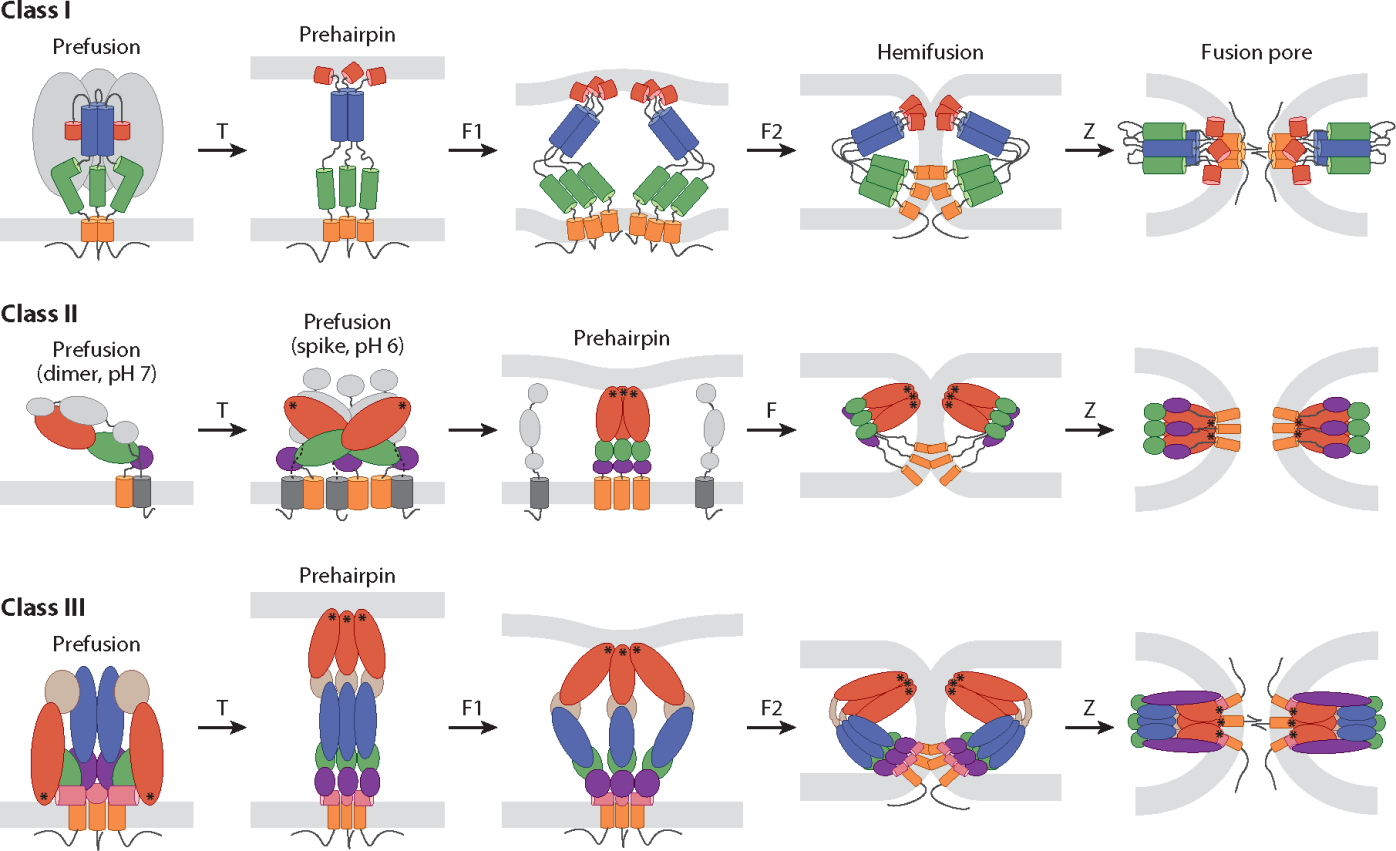
Fusion pathways of class I, II, and III viral fusion proteins. The fusion proteins schematically depicted are human immunodeficiency virus (HIV) envelope (Env) (class I), Semliki Forest virus (SFV) E1 (class II), and herpes simplex virus 1 (HSV-1) gB (class III). In response to specific triggers (T), prefusion structures proceed through stages of extended prehairpin formation, fold-back (F), and zippering (Z) while the membranes progress from separated bilayers through hemifusion and fusion pore formation. F, F1, and F2 denote progressive stages of fold-back but are not meant to imply there are only one or two stages. Color coding and symbols are as follows: Class I: red, fusion peptide; blue, heptad repeat (HR) 1; green, HR2; orange, transmembrane domain (TMD); gray, HIV gp120. Class II, E1: green, domain I; red, domain II; purple, domain III; orange, TMD of E1; asterisks, fusion loops. Class II, (SFV) E2: gray, domains A, B, and C and TMD. Class III: red, domain I; light brown, domain II; blue, domain III; green, domain IV; purple, domain V; pink, membrane proximal external region (MPER); orange, TMD; asterisks, fusion loops. Thin black lines below TMDs denote cytoplasmic tails. See Supplemental Figure Legend 1 for more information and references.

The membrane surrounding each enveloped virus particle sequesters the viral genome. The cell also has a protective membrane barrier. Enveloped viruses breach these barriers simultaneously by fusing the two protective membranes, uniting their previously segregated interiors. Before fusing, viral particles attach to host cell surfaces through attachment factors and/or receptors. For some viruses, the fusion protein also mediates binding to host cells, whereas for others a separate viral protein is employed. After binding to host cells, enveloped viruses then fuse with either the plasma or an endosomal membrane (1) (see the Supplemental Dataset). Fusion proceeds through five key steps in the fusion protein: priming, triggering, extension of a target membrane engaging prehairpin intermediate, fold-back, and zippering. In so doing, the fusion protein accomplishes its mission: overcoming the energy barriers that preclude spontaneous membrane approach and fusion. During these protein dynamics, the fusing bilayers also undergo key changes: close approach, followed by hemifusion and fusion pore formation. While there are different classes of fusion proteins and modes by which they are primed and triggered, a general pathway applies to all (Figure 1).

**Figure 2:**
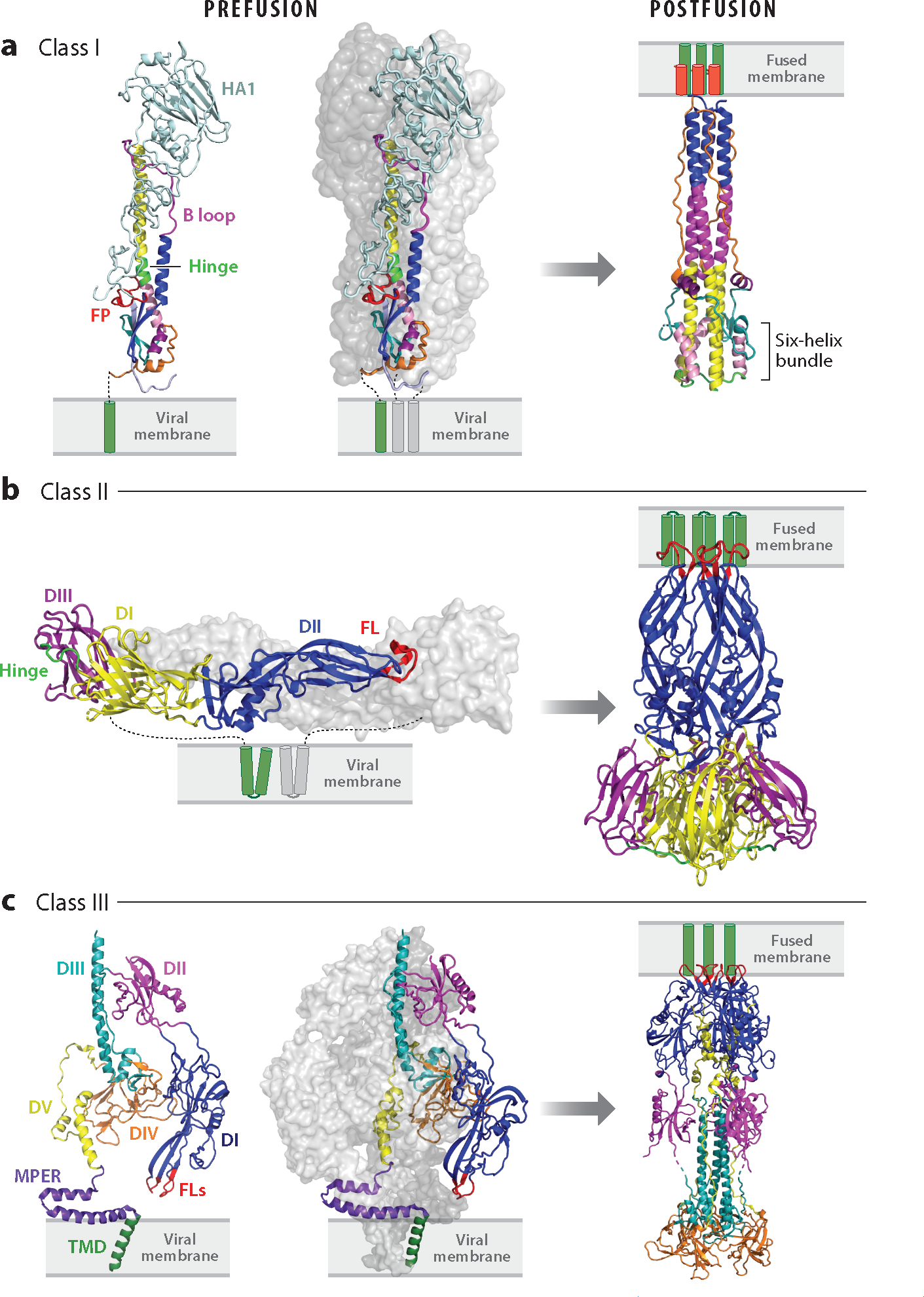
Pre- and postfusion structures of class I, II, and III viral fusion proteins. (*a*) Class I: influenza hemagglutinin (HA) in pre- [Protein Data Bank (PDB) 2HMG] and postfusion (PDB 1QU1) states (*far left*, excised prefusion monomer). The ectodomains of two monomers of the trimer are shown as surface representations in shades of gray; the other is colored: light blue, HA1; red, fusion peptide (FP); blue, helix A; magenta, B loop prefusion and helix B postfusion; yellow, helix C; neon green, helix D prefusion and DE turn postfusion; pink, helix E; teal, loop F; purple, helix G; orange, C-terminal leash. Transmembrane domains (TMDs) are shown in green. HA1 is not seen in the postfusion structure. (*b*) Class II: dengue virus E in pre- (*left*, PDB 4UTB, *side view*) and postfusion (*right*, PDB 1OK8) conformations. In the left panel (prefusion), one E ectodomain monomer is shown in gray and the other is coded with domains I, II, and III in yellow, blue, and purple, respectively; the fusion loops (FLs) are shown in red, and the E TMDs are depicted in green. The companion protein, precursor membrane (prM), is not shown. All class II fusion proteins, including those involved in eukaryotic and archaeal fusion (187, 188), have the same basic architecture. (*c*) Class III: human cytomegalovirus (HCMV) gB is shown in pre- (PDB 7KDP, *left two panels*) and postfusion (PDB 7KDD) conformations; an excised monomer is shown on the far left. Domains I, II, III, IV, and V of one monomer are shown in blue, magenta, teal, orange, and yellow, respectively. The membrane proximal external region (MPER) is in purple, the TMD in green, and the FLs in red. Cytoplasmic tails are not shown in any panels. See Supplemental Figure Legend 2 for more information and additional references.

Each enveloped virus contains a dedicated fusion protein (or complex) (see the Supplemental Dataset). The known viral fusion proteins are all glycosylated type I integral membrane proteins. Their truly unique feature is that they contain two hydrophobic tethers such that once triggered they can be dually anchored in the target and viral lipid bilayers, via fusion peptides/fusion loops and transmembrane domains (TMDs), respectively. For fusion proteins that also mediate cell binding, the two functions reside in separate subunits [e.g., for influenza hemagglutinin (HA)] or domains (e.g., for flavivirus E proteins). Most fusion proteins fall into one of three structural classes (2–6). Class I and II fusion proteins have high contents of alpha-helices and beta-strands, respectively, while class III proteins contain a mix of both (Figure 2). Class I and III fusion proteins are homotrimers both pre- and postfusion. Class IIs are homo- or heterodimers prefusion but enact fusion as homotrimers. The unifying structures are a membrane-embedded prehairpin and a culminating trimer-of-hairpins. There may be other fusion protein classes. Hepatitis C virus (HCV) displays an E1E2 complex. While E1 has a predicted fusion peptide, it displays (7), and E1s of related viruses are predicted to contain (8), a novel fold. Moreover, HCV E2 also appears to interact with the target membrane (9).

## PROTEOLYTIC PRIMING OF VIRAL FUSION PROTEINS

A proteolytic priming step, often mediated by a Golgi-localized furin during virus production, renders class I and many, but not all, class II proteins fusion competent (see the Supplemental Dataset). The critical cleavage event occurs within the fusion protein (class I) or an associated protein (class II). For example, influenza HA0 (class I) is cleaved to HA1 (receptor binding) and HA2 (fusion), which remain disulfide bonded; analogous subunits of human immunodeficiency virus (HIV) envelope (Env) glycoprotein and severe acute respiratory syndrome coronavirus 2 (SARS-CoV-2) spike (S) are held together noncovalently. Arenavirus GPs are primed by cleavage into receptor binding (GP1) and fusion (GP2) subunits, and additionally they contain their signal peptide as a component of their fusion machine (10). Where studied, priming of class I fusion proteins causes only small structural changes. Examples of class II companion proteins that undergo priming cleavages are pE2 proteins of alphaviruses (currently the sole genus in the *Togaviridae* family) and precursor membrane (prM) proteins of flaviviruses (11, 12). Following priming, the associated proteins (class II) or receptor binding domains (class I) prevent premature fusion triggering and therefore potential inactivation in inappropriate cellular locales (4, 12, 13). The priming protease availability can influence cellular tropism and pathogenicity, as well noted for avian influenza viruses (14).

## SITES OF ENVELOPED VIRUS FUSION

Enveloped viruses fuse with either the plasma or an endosomal membrane depending on where appropriate fusion triggers reside (1, 4). The fusion site is usually, but not always, conserved among family members (see the Supplemental Dataset). Generally, parainfluenzaviruses and retroviruses fuse at the plasma membrane at neutral pH following binding to receptors; during infections their fusion proteins can promote cell-cell fusion (syncytia formation), which can affect viral pathogenesis (15–17). Most enveloped viruses, however, are triggered and fuse in endosomes, which become progressively more acidic along the endocytic pathway (4). Dictated by localization of triggering factors, some fuse in early, while others fuse in later, endosomes. Endosomes are heterogeneous in ionic, lipid, and protein compositions as well as location, acidity, and motility; some are not permissive for virus fusion (18). Agents that raise endosomal pH or block endosome maturation or virus trafficking are under consideration as antiviral agents (19). Some viruses have been proposed to employ back fusion to release their genomes into the cytoplasm (20).

### FUSION TRIGGERS

There are four basic means by which fusion proteins are triggered. The trigger employed is generally, but not always, conserved among family members (see the Supplemental Dataset).

1. Low pH, encountered in endosomes, triggers certain class I, II, and III fusion proteins. Examples are influenza HA (class I), dengue virus (DENV) E (class II), and vesicular stomatitis virus (VSV) G (class III). In general, proteins activated from pH ~6 to ~6.8 direct fusion in early endosomes, while ones activated from ~5.0 to ~6.0 do so in later endosomes. Some viruses whose fusion proteins are activated in the early endosome pH range only fuse in later endosomes, which can be attributed to an enhancing role for late endosome (LE)-resident lipids, notably bis(monoacylglycero)phosphate (BMP) (see 21, 22, and references therein). Protonation of histidines (pKa ~6) and in some cases aspartates and glutamates is involved (23–27). In addition to being a trigger, low pH can affect other fusion protein features including fusion peptide/loop structure (28–30) and stability of trimers-of-hairpins (31); a fusion protein’s acid stability can influence virus transmissibility (13, 32).
2. Receptors trigger certain class I and III fusion proteins. A well-explored case is the HIV Env glycoprotein (33). HIV Env first engages CD4 on the surface of target cells. This induces a conformational change that promotes Env binding to a chemokine receptor, CXCR4 or CCR5. CD4 binding can be considered a postproteolytic priming (generates gp120 and gp41) preparatory event, with engagement of the chemokine receptor as the HIV coreceptor that triggers fusion. Of note, presence of the fusion trigger, a chemokine receptor, influences the cellular tropism of HIV. Other retroviral fusion proteins (all class I), including Envs of simian immunodeficiency virus, human T lymphotropic virus, avian sarcoma leukosis virus (ASLV), and murine leukemia virus, are also triggered by their receptors. Activation of those containing both a CXXC and a CX6CC motif appears to involve disulfide exchange following receptor binding (4). Binding to cell surface receptors also triggers the fusion proteins of paramyxoviruses (34), but for these, binding is via a separate viral glycoprotein, alternatively termed H, G, or HN. Upon engagement, the binding protein undergoes a change that is relayed to, and thereby triggers, F (35, 36). Another example of receptor triggering through a relay mechanism is seen for herpesviruses including herpes simplex virus (HSV)-1 and -2, human cytomegalovirus, and Epstein-Barr virus. For these, the fusion protein, gB (class III), is a constituent of a four-component fusion machine that also contains gH/gL and gD (nomenclature for HSV). For HSV-1, receptor binding to gD is relayed through gH/gL, which signals to and thus triggers gB (37–41). Simple receptor-triggered fusion events occur at neutral pH leading to virus entry through the plasma membrane. Low pH-requiring class I fusion proteins from the arenavirus (10, 42, 43), filovirus (44–46) and arterivirus (47) families engage endosomal receptors. For Lassa fever virus (LASV), the endosomal receptor, Lamp1, is not absolutely required but rather upwardly shifts the fusion pH threshold (from ~5.0 to ~5.5), thereby enhancing infection (48). Receptors may also participate in low pH-dependent fusion of HCV and related viruses (9, 49).
3. ASLV Env undergoes a two-step triggering process. Receptor binding induces the fusion subunit (gp37) to form an extended intermediate and insert its fusion loop into the target membrane. Low pH then triggers the fold-back step generating the trimer-of-hairpins that unites the target and viral membranes (4). Another example of two-step fusion may be for rubella virus (RUBV). RUBV E1 has two fusion loops; Ca^+2^ coordinates them to present a hydrophobic surface to the target bilayer (50). This reflects the fact that RUBV, which enters cells through early endosomes, requires Ca^+2^ in addition to low pH for full fusion. In vitro, RUBV particles bind to liposomes at neutral pH in the presence of Ca^+2^, but binding only becomes irreversible if also exposed to low pH; E1 homotrimer formation and fusion also require low pH (51). The composite findings suggest an initial Ca^+2^-dependent target membrane binding step, followed by low pH-dependent refolding steps (52). SARS-CoV-2, the etiologic agent of coronavirus disease 2019 (COVID-19), has also been suggested to undergo a two-step fusion process (53), and HCV fusion appears to involve both receptor binding and low pH (9, 49).
4. The first example of protease-triggered fusion was for the S glycoprotein of severe acute respiratory syndrome coronavirus 1 (SARS-CoV-1) (4, 54, 55). Following binding to its receptor (ACE2), proteases, including cell surface serine proteases such as TMPRSS2, can cleave S, triggering fusion at the plasma membrane. In cells lacking a suitable surface protease, particles enter endosomes and fuse in response to an analogous cathepsin-mediated cleavage. SARS-CoV-2 S is primed by furin in virus-producing cells yielding receptor binding (S1) and fusion (S2) subunits (56). Binding to its receptor (also ACE2) exposes the S2′ site (upstream of a fusion domain), which is then cleaved by cell surface proteases such as TMPRSS2. This is the general scenario in lung epithelial cells, but SARS-CoV-2 can enter other cells through endosomes. The entry route preference differs among SARS-CoV-2 variants dependent on sequences at the S1/S2 (furin site) junction; these differences can influence cellular tropism and transmission potential (54, 55, 57–60). For a murine coronavirus, receptor binding leads to a membrane-embedded prehairpin, with protease cleavage at S2′ instigating the fold-back steps—i.e., is a two-step fusion process (number 26 in Reference 61). A recent study has provided evidence that even in lung cells, SARS-CoV-2 requires endocytosis and mildly low pH (pH 6.8) for infection; the latter is postulated to trigger fold-back of the membrane-embedded S prehairpin. Concordantly, the pH in the human nasal cavity was recorded to be ~6.7 (53).

The GPs of Ebola and other filoviruses are class I fusion proteins that require multiple steps to prepare and trigger them for fusion. They are primed by furin in producer cells generating receptor binding (GP1) and fusion (GP2) subunits. Following attachment to cells and internalization, GP1 is cleaved by endosomal cathepsins generating an ~19-kDa form. The ~19-kDa GP then binds Niemann–Pick C1 (NPC1) (44, 45, 62), which induces changes that likely facilitate release of the fusion loop (63–66). Fusion by ~19kDa GP requires low pH (65) and is inhibited by cathepsin protease inhibitors (67) (number 35 in Reference 67). Low pH alters the structure of the fusion loop (28, 29), allowing it to bind deeply into target membranes (68), and also stabilizes the six-helix bundle in the trimer-of-hairpins (31). Recent work supports the proposal that further cathepsin action provides a final impetus for optimal fusion (69). Optimal fusion also requires Ca^+2^, which affects GP conformational change dynamics (65) and the structure of the fusion loop (70).

## FUSION-INDUCING CONFORMATIONAL CHANGES IN VIRAL FUSION PROTEINS

There is now a wealth of information regarding the pre- and postfusion structures of class I, II, and III fusion proteins (2–6, 11, 12, 71–73) (Figure 2). Recently, intermediate states have come into focus.

### Intermediates in Class I Fusion Proteins

Influenza HA has served as a paradigmatic (low pH-triggered) class I fusion protein (see the Supplemental Dataset). While intermediates between pre- and postfusion HA were previously inferred, recent studies using single-molecule fluorescence resonance energy transfer (smFRET) (64, 74, 75), hydrogen/deuterium exchange mass spectrometry (HDX-MS) (75), cryo–electron microscopy (EM) (75, 76), X-ray crystallography (77–79), and single-particle fusion (80, 81) have illuminated them. Several points have emerged: (*a*) The prefusion structure is not static but reversibly samples conformers more strongly evoked by low pH. (*b*) Three intermediate states have been identified (76). (*c*) Release of the fusion peptide is an early rate-limiting step occurring before significant separation of the globular heads (75, 76, 80), as reported earlier based on antibody binding studies. (*d*) There is functional cross talk between the globular head (HA1) and fusion (HA2) domains (81). (*e*) The head domains separate progressively (76). (*f*) The extending coiled-coil threads through the separating head domains (76) en route to forming the target membrane-embedded prehairpin. (*g*) Changes in linker (sometimes termed hinge) regions are key.(*h*) Multiple activated trimers cluster at the fusion site (2–4, 82, 83). (*i*) The juxta-TMD linker region is flexible, allowing the ectodomain to tilt vis-à-vis the viral membrane, likely facilitating refolding steps (84). (*j*) To complete the fold-back, the C-terminal segment of the ectodomain, sometimes referred to as the leash, packs in the grooves of the long N-helix bundle (85). (*k*) The TMD affects conformational dynamics (75, 76). Intermediates of other class I fusion proteins have been investigated similarly, including smFRET (86) and cryo-EM (83, 87) studies on HIV Env; smFRET studies on Ebola GP (65, 66); and smFRET (88, 89), cryo-EM (90–94), and HDX-MS (95) studies on SARS-CoV-2 S. Although details differ, HA-reminiscent themes are emerging.

### Intermediates in Class II Fusion Proteins

Class II proteins are homo- or heterodimers that are further arrayed in lattices on viral particles (11, 12, 71). For example, E1, the fusion protein of Semliki Forest virus (SFV), is a heterodimer with E2, and they are further arrayed as E1E2 trimeric spikes on virions (Figure 1). In response to low pH, E2 dissociates and E1 engages the target membrane through its fusion loop, first as a monomer. Monomeric E1s then organize as trimers, yielding the common membrane-embedded trimeric prehairpin (71) (Figure 1). This scenario is based on biochemical experimentation. Now, intermediates in several class II proteins have been examined using cryo-EM. Studies on the alphavirus Eastern equine encephalitis virus revealed two states before formation of the E1 homotrimer involving 20° and 60° rotations of the E2 B domain (96). Recent work examined fusion of Chikungunya virus, another alphavirus, with liposomes at low pH by cryo–electron tomography (ET). Timed sampling revealed nine stages reflecting protein (E1E2) and membrane remodeling. Notably, the liposome-embedded E1 homotrimer, hemifusion, and pore formation were visualized (97). Recent cryo-ET work has also observed initial stages of activation for an orthobunyavirus GnGc complex (98).

### Intermediates in Class III Fusion Proteins

The most extensively studied class III fusion proteins are rhabdovirus Gs and herpesvirus gBs; class III fusion proteins are also found in baculo- and thogotoviruses. Rhabdovirus Gs (e.g., VSV G) are activated solely by low pH. Unlike most other virus fusion proteins, their conformational changes are reversed upon returning pH to neutral, i.e., they are not metastable. While homotrimeric both pre- and postfusion, rhabdovirus Gs appear to transit through a monomeric state before forming their postfusion trimers (24, 99–101). The changes in G between pre- and postfusion involve large domain rotations, notably reorienting the fusion loops (at the tip of the fusion domain) from pointing toward the viral membrane to pointing upward to the target membrane; like postfusion class I proteins, rhabdoviral Gs contain a six-helix bundle. On virions, postfusion VSV Gs group in regular arrays outside of the membrane contact zone, which has been speculated to help drive fusion pore expansion (24, 99, 100, 102).

Overall the architectures of pre- and postfusion herpesvirus gBs are similar to those of rhabdovirus Gs (37, 103, 104), with noted exceptions (104, 105). Biochemical and mutagenic, as well as cryo-EM, studies (16, 103, 105) have suggested a pathway of intermediates. As for VSV G, the dramatic change is movement of the fusion domain, reorienting the fusion loops from facing the viral, to facing the target, membrane (Figures 1 and 2). A hinge in domain III with a short motif also seen in rhabdovirus Gs allows this major flip (Figure 2c). It is important to recall that unlike rhabdoviral Gs, which are activated solely by low pH, gBs require partners (gD and gH/gL for HSVs) and receptor binding to enact fusion. gBs are also metastable while rhabdovirus Gs are not. And unlike rhabdovirus Gs, the gB proteins do not appear to transit through a monomeric intermediate. Also of note, the C-terminal end of HSV-1 gB (including its TMD and long cytoplasmic tail) plays an important role in coordinating fusion (37, 104, 106). Clearly, details remain to be discovered as to how the gD-gH/gL-gB fusion machine and other class III fusion proteins function.

## FOCUSING ON THE MEMBRANES: ORGANIZATION, DYNAMICS, AND ENERGETICS DURING FUSION

**Figure 3:**
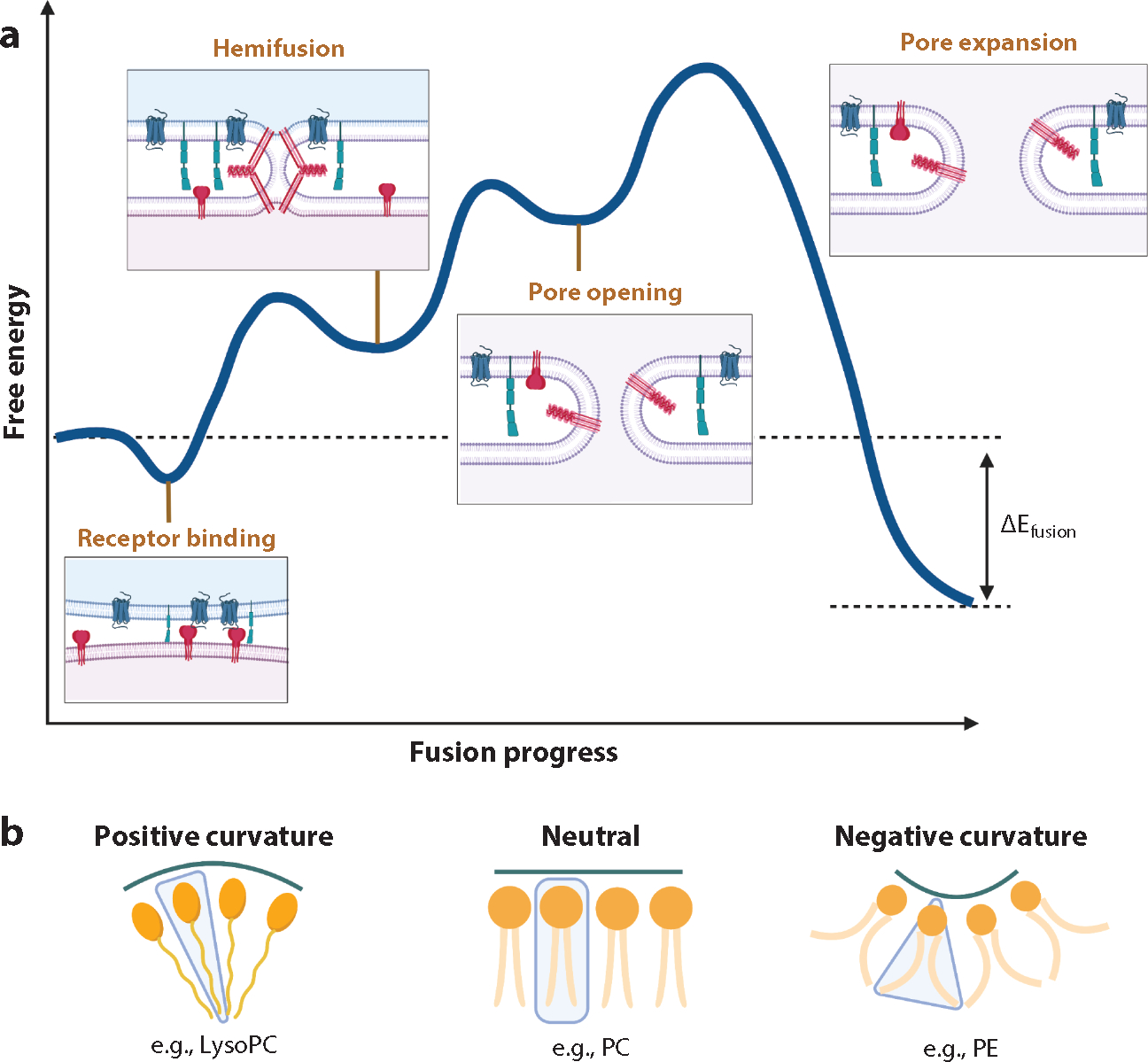
Energetics and membrane dynamics during fusion. (*a*) An approximated schema of fusion energetics as exemplified by the human immunodeficiency virus (HIV) envelope (Env) glycoprotein (protein symbols as in Figure 4). The blue curve represents approximate free energy from receptor binding to hemifusion to pore opening to pore expansion. The energies depicted are approximate, but the transition energies (peaks) range from ~10 to 100 k_B_T. (*b*) Lipids can have positive [lysophosphatidylcholine (lysoPC)], negative [phosphatidylethanolamine (PE)], or no [phosphatidylcholine (PC)] intrinsic membrane curvature. Negative intrinsic curvature of the exterior leaflet stabilizes hemifusion intermediates, and positive intrinsic curvature in the interior leaflets stabilizes fusion pores. Figure adapted from Reference 121 with permission. See Supplemental Figure Legend 3 for more information and references.

The end point of the fusion reaction is energetically downhill from the start but rarely proceeds spontaneously due to higher energy intermediate and transition steps (23, 107) (Figure 3). Initially, fusing membranes must overcome the repulsive hydration force separating the two membranes. Once water has been cleared from the contact zone, dehydrated headgroups of the external leaflets can interact and form a small area of close contact, which is thought to depend on removal of at least one acyl chain from the hydrophobic center of the bilayer toward the polar headgroups (108). With further input of energy to bend membrane leaflets and transiently expose lipid tails to more water, the contact area forms a hemifusion stalk (107) (Figure 3a). This stalk is likely free of transmembrane proteins because hydrophilic cytoplasmic domains thereof would have to transit the hydrophobic bilayer interior of the stalk. Recent work suggests that membrane deformability affects the energy required to reach the hemifusion stage (109). Hemifusion is generally agreed upon as an on-pathway intermediate. It remains undecided, however, whether the vertically oriented hemifusion stalk proceeds directly to an open fusion pore or first widens into a more extended hemifusion diaphragm. The answer may depend on properties of the membranes involved such as lipid composition and curvature (107, 108). Moreover, the forces that create a hemifusion diaphragm may lead quickly to fusion pore opening, as observed in molecular dynamics simulations. During pore opening, the inner leaflets of the two membranes fuse and an aqueous channel opens between the fusing compartments (108) (Figure 3a). In models of pores arising directly from hemifusion stalks, fusion proteins may stabilize the stalk and mechanically drive pore opening, with associations between fusion peptides and TMDs, providing required energy (68, 73, 110). Changes to the underlying viral matrix may assist pore formation or expansion (111–114).

Hemifusion stalks, diaphragms, and fusion pores are highly curved membrane states. Thus, columnar lipids, e.g., phosphatidylcholine (PC), must tilt or splay their acyl chains to accommodate curvature. Tilt applied to columnar lipids creates voids between acyl chains, which incurs an energetic penalty. Lipids with intrinsic curvature such as the cone-shaped phospholipids phosphatidylethanolamine (PE) and lysophosphatidylcholine (lysoPC) with negative and positive spontaneous curvature, respectively, can accommodate curvature without creating voids (Figure 3b). Accordingly, hemifusion intermediates are stabilized by negative spontaneous curvature in the exterior leaflets. In the inner leaflet, negatively curved lipids, such as PE, inhibit fusion pore formation and positively curved lipids, such as lysoPC, promote fusion pores (108).

The phospholipid composition of participating bilayers clearly affects many aspects of fusion (115–117). In addition to general effects of PE and lysoPC on membrane curvature, specific lipids facilitate fusion by certain viruses. As discussed above, BMP, which is enriched in LE, enhances endosomal fusion of influenza A virus, DENV, yellow fever virus, and Japanese encephalitis virus, where studied at the fusion pore stage (21, 22, 115). Similarly, at the plasma membrane, HIV promotes nonapoptotic phosphatidylserine exposure to increase charge on the fusing membrane and decrease some of the energetic cost of headgroup dehydration (117, 118).

**Figure 4:**
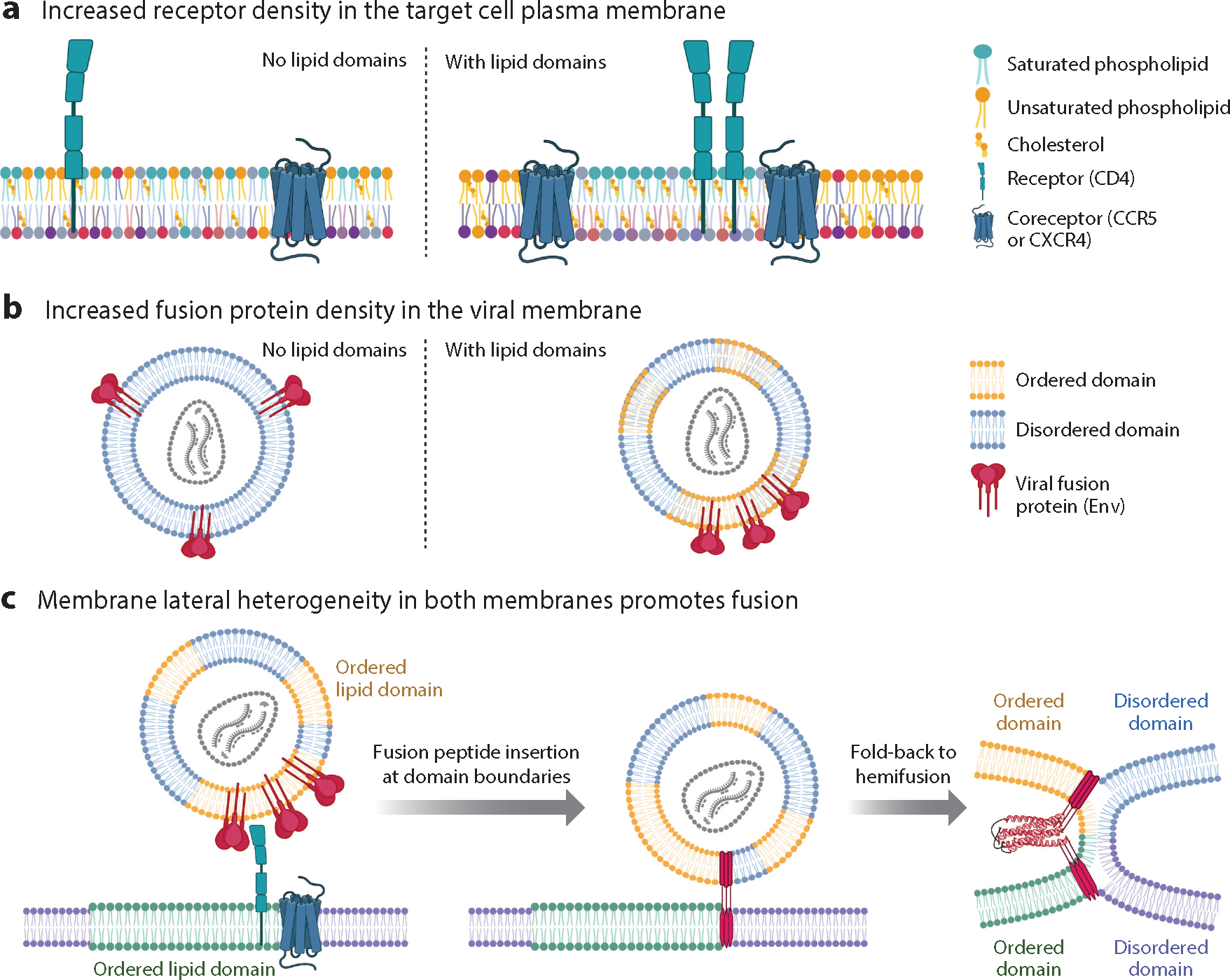
Effects of membrane lateral heterogeneity on viral membrane fusion as exemplified by human immunodeficiency virus (HIV). (*a*) In target cell membranes, lipid nanodomains can organize and concentrate receptors and/or fusion triggers. For HIV, the receptor, CD4, partitions to ordered nanodomains [rich in saturated (*teal*) phospholipids and cholesterol] while the HIV coreceptor, CCR5 (fusion trigger), partitions to the domain boundary. (*b*) In the viral membrane, lipid nanodomains influence the spacing of fusion proteins. On immature HIV particles, envelope (Env) is relatively immobile. Upon maturation via proteolytic cleavage of the juxtamembrane Gag polyprotein, Env diffuses more rapidly to form clusters that facilitate fusion. Env has multiple sequences that promote association with ordered nanodomains (*yellow* phospholipids; cholesterol not depicted) including a cholesterol recognition amino acid consensus motif within the membrane proximal external region (MPER) and palmitoylation sites, and an additional cholesterol interacting domain in the cytoplasmic tail of gp41. Env partitioning to ordered domains might be cell type and HIV strain dependent. (*c*) Lateral heterogeneity affects the energetics of fusion. The fusion peptides in the gp41 prehairpin preferentially insert at discontinuities in bilayer thickness between ordered and disordered lipid nanodomains. Upon fusion, the joining of two ordered domains produces one larger domain with a lower ratio of perimeter/area than the starting smaller domains. This minimizes line tension at the domain boundary and contributes favorably to the energetics of fusion. Figure adapted from images created with BioRender.com. See Supplemental Figure Legend 4 for more information and references.

Fundamental to many effects of lipids on fusion is the concept of lateral membrane organization by coexisting disordered and ordered lipid nanodomains, previously termed lipid rafts (119). Favorable associations between saturated phospholipids (notably sphingomyelin) and cholesterol enrich them in nanometer-scale ordered domains that also minimize unfavorable associations with unsaturated phospholipids, which are primarily found in disordered domains (Figure 4a). Rigid packing of lipids and cholesterol gives ordered domains different physico-chemical properties including increased rigidity and thickness and reduced water penetration and compressibility. Proteins can cooperate with membrane lipids to nucleate, stabilize, or destabilize lipid nanodomains.

Viral membranes are generally enriched in cholesterol and sphingomyelin compared to the host cell membranes from which they bud. This composition along with observed domain formation in viral membrane extracts and the induction of membrane ordering by the fusion peptide and TMD of influenza A has led to proposals in which viral membranes are phase separated (120). With advances in cryo-EM and image processing, membrane thickness variations consistent with ordered and disordered domains have been observed directly in intact HIV particles (121). Functionally, nanodomains could concentrate the ~10 Env trimers on HIV particles into clusters (83, 122), which should facilitate fusion (Figure 4b). Depletion of cholesterol or mutation of Env’s cholesterol-interacting cytoplasmic tail decreases Env clustering and fusion (123), as supported by studies on an isolated membrane proximal external region (MPER)-TMD protein (124). Even for virus particles such as influenza A that are densely covered with fusion proteins, cholesterol depletion modifies their distribution on the virion and fusion in a biphasic manner, which can be explained by HA’s preference for ordered lipid domains (125). Cholesterol is required for fusion of other enveloped viruses including alphaviruses (71), Ebola virus (126), and SARS-CoV-2 (127), although mechanisms may vary. Cholesterol plays multiple roles in eukaryotic cell membranes including regulating fluidity, water penetration, curvature, thickness, compressibility, and nanodomain organization, which can all alter the energetics of fusion (128).

While the influence of cell membrane nanodomains has been explored for many enveloped viruses (117, 128, 129), the most thoroughly studied is regarding HIV. Within phase-separated plasma membranes, the HIV receptor, CD4, preferentially partitions within ordered domains while its coreceptor, CCR5, partitions to the interface of ordered and disordered domains (130) (Figure 4a). Similarly, the HIV fusion peptide preferentially induces fusion at the domain boundary of phase-separated membranes (131). The height discontinuity between ordered and disordered domains may facilitate fusion peptide insertion. Fusion of smaller domains within the HIV membrane with those of the target cell membrane into larger domains (Figure 4c) minimizes line tension and contributes favorably to the energetics of fusion (121, 132, 133). Minimization of line tension and fusion at domain boundaries likely facilitate fusion of other enveloped viruses.

Organizing entry promoting factors in appropriate plasma membrane domains is likely important for other viruses. For example, the SARS-CoV-2 receptor (ACE2) and its fusion triggering protease (TMPRSS2) both appear to reside in ordered domains (117). For Kaposi sarcoma herpesvirus, binding to integrin receptors in disordered domains has been reported to lead to ubiquitination of the integrin cytoplasmic tails and consequent relocation to ordered domains (number 74 in Reference 117). Viruses can also alter the host membrane around their binding site as exemplified by measles virus; receptor binding activates host sphingomyelinases to convert sphingomyelin to ceramides, which promotes relocation of CD150 to the plasma membrane and enhances fusion (numbers 75 and 76 in Reference 117). Of note, glucosylceramide (134) and sphingomyelin (135) have been shown to promote entry of several endosome-entering viruses.

## MEMBRANE INTERACTING SEGMENTS OF VIRAL FUSION PROTEINS: PULLING IT ALL TOGETHER

**Figure 5:**
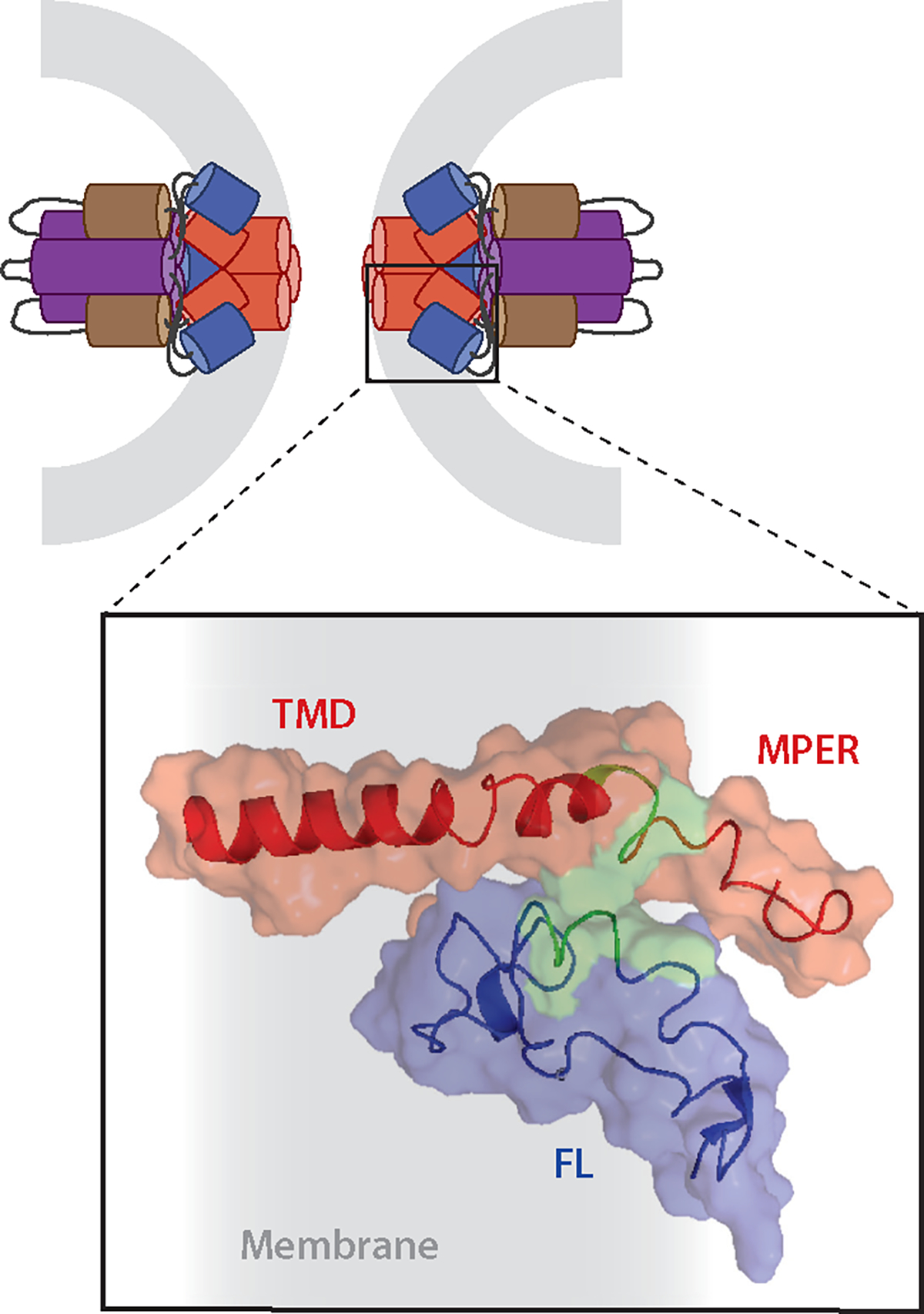
A complex between fusion peptides/fusion loops (FLs) and membrane proximal external region (MPER)-transmembrane domain (TMD) regions completes the fusion protein refolding process that accompanies fusion pore opening. (*top*) Illustration of the fusion pore stage of Ebola virus glycoprotein (GP) mediated fusion; only GP2 is shown, and it is color coded: purple and brown, respectively, the N- and C-terminal heptad repeats; blue, FL; red, MPER-TMD. Gray represents the recently merged membrane. (*bottom*) Blowup of the boxed region in which the nuclear magnetic resonance (NMR) structures in membrane mimetics of the Ebola virus FL (*blue*) and MPER-TMD (*red*) were docked based on experimental interaction constraints from fluorescence and NMR data. Green denotes interacting residues. Gray represents membrane. Figure adapted from Reference 68. See Supplemental Figure Legend 5 for more information and additional references.

The membrane interacting segments of viral fusion proteins—fusion peptides/loops, MPERs, and TMDs—are key to productive fusion. Cytoplasmic tails can also play a role by modulating fusion protein localization and conformational changes (73). Fusion peptides/loops and TMDs dually anchor fusion proteins in the target and viral membranes, respectively, commencing at the prehairpin stage and their union, along with MPERs, helps form the tightly composed trimer-of-hairpins that executes the final steps of fusion (Figures 1 and 5).

### Fusion Peptides, Loops, and Patches

All studied viral fusion proteins contain a fusion peptide or fusion loop(s), moderately hydrophobic sequences that engage the target lipid bilayer once the protein changes conformation in response to its fusion trigger(s). The fusion peptides of most class I fusion proteins are ~20–25 residues long and located at the extreme N terminus of the fusion subunit. For example, the influenza HA and HIV Env fusion peptides comprise the first ~23 and ~20 residues, respectively, of HA2 and gp41 (30, 33, 136–138). Fusion peptides are often enriched in glycines and alanines, which endows them with rich conformational flexibility and structural polymorphism that depends on specific lipid, pH, and protein environments. For example, the HIV Env fusion peptide structure is fungible in terms of alpha-helical and beta-sheet structure, depending on the content of cholesterol (138). Some class I fusion proteins contain fusion loops. For example, the fusion subunit of ASLV contains a 37-residue fusion loop bounded by two disulfide-bonded cysteines, and Ebola virus GP2 harbors a 46-residue fusion loop that is also closed at its ends by a disulfide bond (28, 29). Fusion peptides and loops of pH-triggered class I fusion proteins can change conformation significantly upon pH lowering (2, 28, 29, 139), consolidating hydrophobic residues into hydrophobic patches that facilitate membrane insertion. The hydrophobic patches also promote fusion peptide self-association, which may aid the initiation of fusion pore formation (140). Small motifs of bulky hydrophobic and aromatic residues, parts of the consolidated hydrophobic patches, are often conserved in fusion peptides from the same virus family. Molecular dynamics simulations show that lipid bilayers become partially dehydrated and lipids adapt to the shapes of the inserted peptides in their immediate vicinity resulting in bilayer thinning (140, 141).

Class II fusion proteins have more rigid fusion loops that are pre-formed at the tips of domain II of their fusion subunits. For most, these fusion loops are protected by interactions with the receptor binding subunits or domains in the native dimers (12). Upon triggering, the dimers dissociate and three fusion subunits unite to present a hydrophobic patch at the tip of the trimer (71). The structures of the individual fusion loops of most class II fusion loops are thought to not change much upon interaction with the target membrane. This differs for RUBV E1, which was the first fusion protein (a class II) shown to require Ca^+2^; Ca^+2^ coordinates its two fusion loops, altering the tip structure and thereby increasing the hydrophobicity of the membrane interacting surface and enabling fusion (51, 52). Several class II fusion proteins have been shown to require cholesterol for fusion loop insertion into target membranes (71, 142).

The class III VSV G and herpesvirus gB fusion proteins have two small fusion loops at the ends of the beta-sheets of their fusion domains. These contain key aromatic residues (two tyrosines and a tryptophan for VSV G) that upon triggering present a hydrophobic surface to the target membrane (24, 37, 99, 104). Interestingly, for herpesvirus gBs, the MPER is thought to preclude fusion loop interaction with the viral membrane (Figure 2c) prior to triggering (103, 143).

### Membrane Proximal External Regions, Stems, and Transmembrane Domains

MPERs are mostly amphipathic linker regions located between a fusion protein’s ectodomain and its TMD. Based on structural studies, MPERs have been reported for members of all classes of fusion proteins, including HIV Env (class I), a flavivirus E (class II), and HSV-1 gB (class III) (12, 68, 73, 144). Mutagenesis studies on multiple viral fusion proteins have also attributed functionally important roles for these juxta-viral membrane regions (2, 73, 143, 145, and references therein). A study on HSV-1 gB suggested that its MPER modulates the ability of its fusion loop to interact with target bilayers (2, 143). Flexibility of MPER regions can also afford orientational freedom such that fusion proteins can locate and bind receptors (through receptor binding domains), as described for HIV Env and SARS-CoV-2 S (146, 147). MPER flexibility, altering the ectodomain tilt with respect to the viral membrane, has also been seen for influenza HA (84). Flexibility of juxtamembrane linkers likely also allows the fusion protein to accommodate the highly curved membranes of fusion intermediates.

The TMDs of viral fusion proteins are key players (73). Their physico-chemical properties including length, hydrophobicity, and lipid accessible surface area must be compatible with the different membranes in which TMDs reside at different stages of the viral life cycle (148). Like those of most membrane proteins, and like their fusion peptides/loops, viral fusion protein TMDs influence and are influenced by their surrounding lipid environment (119); most have been reported to be alpha-helical. Consistently, a cryo-EM study of full-length influenza HA in detergent micelles revealed an alpha-helical structure. Interestingly however, two forms were observed. In one, the three TMD helices align with the ectodomain trimeric axis; in the other, they are rotated vis-à-vis one another, perhaps reflecting different organization at different stages of fusion (84). Heterogeneity in angles of TMD membrane penetration might cause membrane thinning, which would promote fusion (149). Non-helical TMD structures have also been reported. For example, the isolated human parainfluenza virus 5 (HPIV5) TMD is reported as alpha-helical in phosphocholine and phosphoglycerol membranes but to contain beta-sheet elements in membranes enriched in phosphoethanolamine. The beta-sheet conformation induced membrane curvature and lipid splay, which are predicted to promote hemifusion (150). While mainly alpha-helical, the C-terminal end of the HIV Env TMD adopts some beta structure in membranes containing 30% cholesterol (144). In this context it is interesting that many viral fusion protein TMDs or cytoplasmic tails have cholesterol binding motifs (68, 126, 151–154). These, along with acylation of C-terminal domains, contribute to localization of some fusion proteins in cholesterol-enriched nanodomains (155). Mutating these motifs has profound effects on membrane fusion, although proposed mechanisms vary.

It has long been postulated that complex formation between fusion peptides/loops and TMDs would represent a final zippering stage in forming the trimer-of-hairpins (Figure 1). Recently it has been shown that MPERs, in some cases along with their TMDs, interact with their respective fusion peptides (68, 73, 110), as modeled for the Ebola GP complex (Figure 5). Packing of C-terminal ectodomain leashes for influenza HA (85) and stems for class II fusion proteins (5, 142) and herpesvirus gBs (103, 105) into their N-terminal trimeric cores has been reported as part of the final zippering stage for some viral fusion proteins. This would facilitate the ultimate joining of fusion peptides/loops and MPERs-TMDs.

### HOST RESTRICTION OF VIRAL FUSION

It is well established that host cells deploy restriction factors to temper postentry steps of viral replication. What has come to light in recent years is that there are also restriction factors that inhibit the fusion step employed by enveloped viruses (156–158).

### Interferon-Inducible Transmembrane Proteins

Interferon (IFN)-inducible transmembrane proteins (IFITMs) inhibit entry of enveloped viruses containing class I, II, or III fusion proteins including HIV, influenza A, SARS-CoV-2, West Nile virus, DENV, and VSV (159). Comprising three antiviral isoforms (IFITM1-3), these IFN-stimulated proteins increase host cell membrane order by modulating membrane curvature via an amphipathic helix, destabilizing the hemifusion state and preventing progression to full fusion (160). Generally, IFITM1 localizes to the plasma membrane while IFITM2 and IFITM3 localize to endosomes, explaining some of the isoform specificity of the viruses they restrict. Additionally, IFITMs can incorporate into viral particles during budding and decrease infectivity of those particles. IFITMs form homomultimers as well as complexes with other host proteins including the zinc metalloproteinase STE24, which potentiates the antiviral effects of IFITM3 and can independently restrict viral entry via an incompletely understood mechanism (157, 161).

### Serincs

The Serinc family is composed of five paralogues. Initially identified as retroviral restriction factors, they are now known to inhibit diverse enveloped viruses including influenza A, SARS-CoV-2, and hepatitis B viruses (162–166). Serinc3 and Serinc5 localize to the plasma membrane. In the absence of viral antagonism, they incorporate into budding HIV particles and decrease their ability to infect subsequent cells by inhibiting membrane fusion at several intermediate steps (167). While Serinc5 does not alter the lipid composition of the HIV viral membrane, more recent studies have demonstrated that Serinc3 and Serinc5 flip phospholipids across leaflets of the viral membrane (168) and that Serinc5 alters membrane lateral heterogeneity of HIV viral particles (121). These changes to the viral membrane likely disfavor fusion and destabilize Env. Serinc5’s effects on viral membrane organization could induce the noted effects on Env or may be independent means of fusion inhibition.

### Cholesterol-25-Hydroxylase

Cholesterol-25-hydroxylase (CH25H) is an IFN-induced enzyme that resides within the endoplasmic reticulum and catalyzes the oxidation of cholesterol to 25-hydroxycholesterol (25HC), which inhibits viral fusion by modifying cellular membranes as well as by initiating a transcriptional program that inhibits cholesterol biosynthesis. As 25HC is secreted, these effects extend to neighboring cells in a paracrine manner (157).

## THERAPEUTIC RESTRICTION OF VIRAL FUSION

There are also human-made means to thwart viral fusion. One is to deploy monoclonal antibodies (mAbs). While many therapeutic mAbs target virus binding to host cells, fusion-blocking mAbs have also been described (50, 66, 84, 169–173). Broadly neutralizing antibodies have also been identified that target MPERs including ones against HIV Env (174, 175), filovirus GPs (176), influenza HA (177), and SARS-CoV-2 (178). MPER-targeting mAbs restrict flexibility, limiting the range of motion of fusion protein ectodomains (84, 175). Peptides can also block fusion, as pioneered by studies of T20, a peptide mimetic of the heptad repeat 2 region of HIV Env gp41. By binding to the heptad repeat 1 coiled-coil, T20 blocks the fold-back step of fusion (Figure 2). Analogous peptide inhibitors have been described for other class I fusion proteins, including against paramyxovirus F proteins (179) and SARS-CoV-2 S (180–182). Peptide mimetics of broadly neutralizing stem antibodies have also been designed (183). Small molecules that disrupt fusion by targeting either host factors or viral fusion machinery are also under consideration (19). Notably, several class I fusion proteins have pockets that, when filled with a small molecule, prevent fusion-related conformational changes (184, 185). Small molecules have also been identified that thwart fusion by binding to MPERs (186) or to sites targeted by broadly neutralizing mAbs (170).

## Supplementary Material

Supplementary Dataset

Supplementary Figure Legends

## Abbreviations

Prehairpin intermediate: a unifying intermediate for all viral fusion proteins in which the fusion protein has extended and embedded its fusion peptide/loop into the target membrane
Hemifusion: the stage when the outer (*cis*) leaflets have mixed but the inner (*trans*) leaflets remain separated
Fusion pore: after hemifusion, the *trans* leaflets merge and generate an aqueous connection between fusing compartment interiors; initially small, fusion pores expand for productive viral entry
Fusion peptide: a relatively apolar segment (~20 residues) at the amino-terminal end of a fusion subunit that interacts with the target membrane at the extended intermediate prehairpin stage
Fusion loop: a relatively apolar segment (~20–25 residues) found internal to a fusion protein/subunit that interacts with the target membrane at the extended intermediate prehairpin stage; also termed internal fusion peptide
Trimer-of-hairpins: the unifying postfusion structure for all viral fusion proteins
Back fusion: a virus first fuses with intralumenal (intraendosomal) vesicles; fusion of intralumenal vesicles with the limiting endosomal membrane releases the viral genome into the cytoplasm
Bis(monoacylglycero) phosphate (BMP): this late endosome-resident lipid has also been termed lysobisphosphatidic acid (LBPA)
Endosomal receptor: upon reaching endosomes, several viruses employ receptors therein: LASV, Lamp1; Lujo virus, CD63; lymphocytic choriomeningitis virus, CD164; simian hemorrhagic fever virus, CD163; filoviruses, NPC1
Full fusion: the stage when a fusion pore has formed
Six-helix bundle: a common element in postfusion structures of all class I and some class III fusion proteins; for class I proteins, C helices (e.g., heptad repeat 2 for retroviruses) pack in grooves of a triple coiled-coil of N helices (e.g., heptad repeat 1 for retroviruses)
Single-molecule fluorescence resonance energy transfer (smFRET): two fluorescent probes capable of FRET are engineered into a protein at defined sites and monitored for interaction (e.g., in a fusion protein, upon triggering)
Hemifusion stalk: also termed lipid stalk; first connection between *cis* leaflets of fusing membranes in a small contact zone; *trans* leaflets remain separated
Hemifusion diaphragm: connection between *cis* leaflets of fusing membranes over a greater distance than a hemifusion stalk; *trans* leaflets remain separate
Lipid nanodomain: a dynamic association of phospholipids, including sphingomyelins with saturated chains, and cholesterol with distinct physico-chemical properties from the surrounding membrane
Membrane proximal external region: segment with membrane interacting properties found just upstream of the transmembrane domain of a fusion protein
