# Supplementary material for: Viral Membrane Fusion: A Dance Between Proteins and Lipids
Source: Annu Rev Virol. Author manuscript; Available in PMC 2024 Feb 14. (PMC10866366; doi:10.1146/annurev-virology-111821-093413)
Supplement: Supplementary Figure Legends [file NIHMS1963193-supplement-Supplementary_Figure_Legends.pdf]

Supplemental Material: Annu. Rev. Virol. 2023. 10:139-161
https://doi.org/10.1146/annurev-virology-111821-093413
Viral Membrane Fusion: A Dance Between Proteins and Lipids
White, Ward, Odongo, Tamm

Supplemental Figure Legends

Figure 1. Fusion pathways of class I, II and III viral fusion proteins. The fusion proteins schematically depicted are HIV Env (class I), Semliki forest virus (SFV) E1 (class II), and herpes simplex virus-1 (HSV-1) gB (class III). Subsequent to priming (for class I and II proteins; not depicted), and in response to specific triggers (T), the pre-fusion structures proceed through stages of extended pre-hairpin formation, fold-back (F), and zippering (Z) while the membranes progress from separated bilayers through hemifusion, fusion pore formation, and (not shown) fusion pore expansion. F, F1 and F2 denote progressive stages of fold-back; these designations are not meant to infer that there are only one or two stages of fold-back. Some pre-fusion states have been shown to be ensembles of structures, for examples, see References (1–5). For class I and III proteins, triggering involves 'cross-talk' between the receptor binding head and the stalk regions (6–13) (2, 12–14). For class I proteins without a disulfide bond between the receptor binding and fusion subunits (e.g., HIV Env as depicted (gp120, gray) and SARS-CoV-2 S), the receptor binding subunit is shed (15, 16); for others (e.g., influenza HA), the head domains move away from the trimer axis. It has been suggested that class I fusion proteins form membrane-embedded trimeric pre-hairpins in a sequential asymmetric manner (17–19), perhaps with insertion of one fusion peptide as a first step. For an avian retroviral Env and HIV Env, glimpses of membrane-embedded pre-hairpins have been observed by cryo-EM (20, 21). On mature virions at neutral pH, the SFV fusion protein (class II) is a heterodimer of E1 (colored subunit) and E2 (gray subunit), as depicted in the far-left image, that are further arrayed as trimeric spikes. In response to the fusion trigger, low pH (~pH 6), the B domain of E2 is dislodged revealing the fusion loops (asterisks) on E1 (second image), E2 dissociates, and E1 forms a membrane-embedded pre-hairpin. Where studied, target membrane interaction is first mediated by an E1 monomer (not shown) prior to coalescing to the (membrane-embedded) trimeric pre-hairpin (22). See main text and (23–26) for how class III fusion proteins are triggered and change conformation. Color-coding: Class I: red, fusion peptide; blue, heptad repeat 1 (HR1); green, HR2; orange, transmembrane domain (TMD); gray, HIV gp120. Class II: E1: green, domain I; red, domain II; purple, domain III; orange, TMD of E1, asterisk, fusion loop; (SFV) E2: gray, domains A, B, C and TMD. Class III: red, domain I; light brown, domain II; blue, domain III; green, domain IV; purple, domain V; pink, membrane proximal external region (MPER); orange, TMD; asterisks, fusion loops. The orientation is target membrane (light gray) on top and viral membrane (light gray) below. Thin black lines below TMDs denote cytoplasmic tails. Other notes: Flexibility in MPERs, located just prior to TMDs, can be required for foldback stages (27). The HIV peptide inhibitor T20 prevents fusion by blocking six-helix bundle formation (28). Fusion pore formation and/or fusion pore expansion (not depicted) may involve changes to matrix layers underlying the viral membrane (29–31). Eukaryotic and archaeal fusion proteins display structural similarity to class II viral fusion proteins (32, 33). The overall Figure layout is similar to Fig. 1 in (34), with the class II pathway stylized after Fig. 3 in (22).

Figure 2. Pre- and post-fusion structures of class I, II, and III viral fusion proteins. For more details on the pre- and post-fusion structures of viral fusion proteins see (22–25, 35–40) (41). Pre-fusion, Class I and III proteins project perpendicularly, whereas Class IIs lie parallel, to the viral membrane. All viral fusion proteins are projecting trimers in their post-fusion states. Fusion peptide/loop sequences can be found at https://viralfp.bio.di.uminho.pt/. As discussed in the text, intermediates between the pre- and post-fusion forms have recently been detected by advanced biophysical techniques.

(A) Class I: influenza HA (from X:31 influenza A virus; subtype H3N2) in pre- (PDB 2HMG) and post- (PDB 1QU1) fusion states (excised monomer is shown on the far left). The ectodomains of two monomers of the trimer are shown as surface representations in shades of gray, the other is colored as follows with nomenclature as per Ni et al. (42): light blue, HA1; red, fusion peptide (FP); blue, helix A; magenta, B loop pre-fusion and helix B post-fusion; yellow, helix C (invariant helix as per Benton (6)); neon green, hinge region prefusion (referred to in Ni et al. as D-helix) and DE turn postfusion; pink, helix E; teal, loop F; purple, helix G; orange, C-terminal leash (referred to in Ni et al. as C-terminal fragment); green denotes TMDs. HA1 is not seen in the postfusion structure. An evolutionary analysis of the fusion subunits of class I fusion proteins has recently been presented (43). As depicted in Figure 1a, receptor binding subunits (e.g., HIV gp120 and influenza HA1) are said to 'clamp' the fusion protein in an inactive state. Upon triggering, the clamp is released, allowing conformational changes to proceed. For HA, key changes include the B-loop to helix (magenta) transition, helix D (neon green) to loop transition, and packing of the C-terminal 'leash' (orange) in the grooves of the central N-terminal coiled-coil (44–47). Structures of the TMD of an H1N1 influenza HA in detergent micelles were visualized by cryo-EM revealing both straight and tilted TMD helices in trimeric bundles (27).

(B) Class II: Dengue virus type 2 protein E is shown in pre- (left, PDB: 4UTB, side view) and post-fusion (right, PDB: 1OK8) conformations. On virions, dengue E is present as a homodimer that is further arrayed along with its associated protein (prM; not shown) on the virion surface (48, 49) (41). In the left panel (pre-fusion), one E ectodomain monomer is shown in gray and the other is coded with Domains I, II, and III in yellow, blue and purple, respectively; the fusion loops at the tips of beta-strands comprising domain II are shown in red, and the TMDs are depicted in green. Some class II proteins (those of alpha- and flaviviruses) have one fusion loop; some in the *Bunyavirales* order have 2 or 3 fusion loops. For dengue virus E, packing of stem regions into the 'seam' of the trimer has been shown to be important (50, 51). All class II fusion proteins, including those involved in eukaryotic cell-cell fusion and archaeal fusion (32, 33), have the same basic 3-domain architecture, The companion (associated) proteins to the class II viral fusion proteins (prM for flaviviruses (not shown)) are also structurally conserved (52). Class II fusion proteins have been observed in arrays post fusion (53–55).

(C) Class III: HCMV gB is shown in (left 2 panels) in pre- (PDB: 7KDP) and (far right) post-fusion (PDB: 7KDD) conformations; an excised monomer is shown on the far left. Domains I, II, III, IV and V are shown in blue, magenta, teal, orange, and yellow, respectively. The MPER is shown in purple, the TMD in green and the fusion loops (FLs) in red. HMV gB also has a long cytoplasmic tail (~130 residues, not shown). Rhabdoviral Gs have similarly constituted regions corresponding to domains I-IV (56). All class III proteins display fusion loops at the tips of a beta-stranded domain (Domain I for gB; FD (fusion domain) for VSV G). A hinge (between DII and DIII for gB) allows the ~180° flip (of DI and DII) that positions the fusion loops for insertion into the target membrane (56, 57). Some herpesvirus gBs are cleaved by furin (e.g., EBV gB), but others are not (e.g., HSV-1 gB). Nonetheless, gBs are considered to be metastable as they rapidly transit to their post-fusion structures. In contrast, rhabdovirus Gs are not primed and are not metastable; they reversibly sample post-fusion structures upon pH modulation. For rhabdovirus Gs studied, evidence suggests that the extended (target membrane-embedded) intermediate is a monomer that reassembles into a trimer as fusion progresses (5, 26, 58–60). There is no evidence that herpesvirus gBs (or viral membrane anchored class I proteins) are monomers as they insert into the target membrane (though, as stated above, fusion peptides/loops may insert sequentially). In contrast to

rhabdoviral Gs, herpesvirus gBs work in concert with additional proteins to elicit fusion (see main text). Class III fusion proteins have been seen in (altered) arrays post fusion (59, 61, 62).

Figure 3. Energetics and membrane dynamics during fusion. (**A**) An approximated schema of fusion energetics as exemplified by the HIV Env glycoprotein (protein symbols as in the key to Figure 4). The blue curve traces the approximate free energy from receptor binding (slightly negative due to the enthalpy of gp120/CD4 binding), to hemifusion (a higher energy intermediate and local minimum), to pore opening and finally through pore expansion to the ultimately lower energy post-fusion membrane. The energies depicted are approximate but the transition energies (the peaks) range from ~10-100$k_B T$ (47, 63, 64). (**B**) Lipids can have positive (lysophosphatidylcholine, (lysoPC)), negative (phosphatidylethanolamine (PE)) or no (phosphatidylcholine (PC)) intrinsic membrane curvature. Negative intrinsic curvature of the exterior leaflet stabilizes hemifusion intermediates and positive intrinsic curvature in the interior leaflets stabilizes fusion pores.

Figure 4. Effects of membrane lateral heterogeneity on viral membrane fusion as exemplified by HIV. (**A**) In target cell membranes, lipid nanodomains can organize and concentrate receptors and/or fusion triggers. For HIV, the receptor, CD4, partitions to ordered nanodomains (rich in saturated (teal) phospholipids and cholesterol) while the co-receptor, CCR5, partitions to the domain boundary as described in (65). (**B**) In the viral membrane, lipid nanodomains influence the spacing of fusion proteins. On immature HIV particles, Env is relatively immobile. Upon maturation via proteolytic cleavage of the juxtamembrane Gag polyprotein, Env diffuses more rapidly to form clusters that facilitate fusion (66, 67). Env has multiple sequences that promote association with ordered nanodomains (yellow phospholipids; cholesterol not depicted) including a cholesterol recognition amino acid consensus (CRAC) motif within the MPER (68), and palmitoylation sites and an additional cholesterol interacting domain within the C-terminal cytoplasmic tail of gp41 (69). While Env generally partitions to ordered domains, this may be cell type and HIV strain dependent (68). (**C**) In addition to concentrating receptors and fusion proteins, lateral heterogeneity affects the energetics of fusion. After one or more Env trimers bind receptor and co-receptor, the fusion subunit, gp41, forms a pre-hairpin with fusion peptides preferentially inserted at the discontinuity in bilayer thickness between ordered and disordered lipid nanodomains as described in (70). Fold-back to the trimer-of-hairpins promotes hemifusion, fusing the outer bilayer leaflets. Joining two ordered domains produces one larger domain upon full fusion with a lower ratio of perimeter/area than the starting smaller domains. This minimizes line tension at the domain boundary and contributes favorably to the energetics of fusion as described in (71).

Figure 5. A complex between fusion peptides/loops and MPER-TMD regions completes the fusion protein refolding process that accompanies fusion pore opening. Shown on top is a cartoon of the fusion pore stage of ebolavirus GP mediated fusion; only GP2 is shown and it is color-coded: purple and brown, respectively, the N-and C-terminal heptad repeats; blue, fusion loop; red, MPER-TMD. Gray represents the recently merged membrane. Below is a blow up of the region boxed in the cartoon in which the NMR structures in membrane mimetics of the ebolavirus fusion loop (blue) (72, 73) and MPER-TMD (red) (74) were docked based on experimental interaction constraints from fluorescence and NMR data. Green denotes interacting residues. Gray represents membrane. Figure adapted from (74). A recent study presents a cryo-EM

structure showing a complex between the fusion loop and TMD of the post-fusion SAR-CoV-2 S protein (75).

Bibliography

1. Costello SM, Shoemaker SR, Hobbs HT, Nguyen AW, Hsieh C-L, et al. 2022. The sars-cov-2 spike reversibly samples an open-trimer conformation exposing novel epitopes. *Nat. Struct. Mol. Biol.* 29(3):229–38
2. Díaz-Salinas MA, Li Q, Ejemel M, Yurkovetskiy L, Luban J, et al. 2022. Conformational dynamics and allosteric modulation of the sars-cov-2 spike. *Elife*. 11:
3. Lu M, Uchil PD, Li W, Zheng D, Terry DS, et al. 2020. Real-time conformational dynamics of sars-cov-2 spikes on virus particles. *Cell Host Microbe*. 28(6):880–891.e8
4. Lu M, Ma X, Castillo-Menendez LR, Gorman J, Alsahafi N, et al. 2019. Associating hiv-1 envelope glycoprotein structures with states on the virus observed by smfret. *Nature*. 568(7752):415–19
5. Baquero E, Albertini AA, Raux H, Abou-Hamdan A, Boeri-Erba E, et al. 2017. Structural intermediates in the fusion-associated transition of vesiculovirus glycoprotein. *EMBO J.* 36(5):679–92
6. Benton DJ, Gamblin SJ, Rosenthal PB, Skehel JJ. 2020. Structural transitions in influenza haemagglutinin at membrane fusion ph. *Nature*. 583(7814):150–53
7. Benhaim MA, Mangala Prasad V, Garcia NK, Guttman M, Lee KK. 2020. Structural monitoring of a transient intermediate in the hemagglutinin fusion machinery on influenza virions. *Sci. Adv.* 6(18):eaaz8822
8. Das DK, Govindan R, Nikić-Spiegel I, Krammer F, Lemke EA, Munro JB. 2018. Direct visualization of the conformational dynamics of single influenza hemagglutinin trimers. *Cell*. 174(4):926–937.e12
9. Sengar A, Cervantes M, Kasson PM. 2022. Mechanistic dissection of antibody inhibition of influenza entry yields unexpected heterogeneity. *Biophys. J.*
10. Schapiro HM, Khasnis MD, Ahn K, Karagiaridi A, Hayden S, et al. 2022. Regulation of epitope exposure in the gp41 membrane-proximal external region through interactions at the apex of hiv-1 env. *PLoS Pathog.* 18(5):e1010531
11. Das DK, Bulow U, Diehl WE, Durham ND, Senjobe F, et al. 2020. Conformational changes in the ebola virus membrane fusion machine induced by ph, ca2+, and receptor binding. *PLoS Biol.* 18(2):e3000626
12. Benton DJ, Wrobel AG, Xu P, Roustan C, Martin SR, et al. 2020. Receptor binding and priming of the spike protein of sars-cov-2 for membrane fusion. *Nature*. 588(7837):327–30
13. Xu C, Wang Y, Liu C, Zhang C, Han W, et al. 2021. Conformational dynamics of sars-cov-2 trimeric spike glycoprotein in complex with receptor ace2 revealed by cryo-em. *Sci. Adv.* 7(1):
14. Meng B, Datir R, Choi J, CITIID-NIHR Bioresource COVID-19 Collaboration, Bradley JR, et al. 2022. SARS-cov-2 spike n-terminal domain modulates tmprss2-

dependent viral entry and fusogenicity. *Cell Rep.* 40(7):111220
15. Jackson CB, Farzan M, Chen B, Choe H. 2021. Mechanisms of sars-cov-2 entry into cells. *Nat. Rev. Mol. Cell Biol.* 23(1):3–20
16. Lu M, Ma X, Reichard N, Terry DS, Arthos J, et al. 2020. Shedding-resistant hiv-1 envelope glycoproteins adopt downstream conformations that remain responsive to conformation-preferring ligands. *J. Virol.* 94(17):
17. Kawase M, Kataoka M, Shirato K, Matsuyama S. 2019. Biochemical analysis of coronavirus spike glycoprotein conformational intermediates during membrane fusion. *J. Virol.* 93(19):
18. Sjöberg M, Löving R, Lindqvist B, Garoff H. 2017. Sequential activation of the three protomers in the moloney murine leukemia virus env. *Proc. Natl. Acad. Sci. USA*. 114(10):2723–28
19. Riedel C, Vasishtan D, Siebert CA, Whittle C, Lehmann MJ, et al. 2017. Native structure of a retroviral envelope protein and its conformational change upon interaction with the target cell. *J. Struct. Biol.* 197(2):172–80
20. Ladinsky MS, Gnanapragasam PN, Yang Z, West AP, Kay MS, Bjorkman PJ. 2020. Electron tomography visualization of hiv-1 fusion with target cells using fusion inhibitors to trap the pre-hairpin intermediate. *Elife*. 9:
21. Cardone G, Brecher M, Fontana J, Winkler DC, Butan C, et al. 2012. Visualization of the two-step fusion process of the retrovirus avian sarcoma/leukosis virus by cryo-electron tomography. *J. Virol.* 86(22):12129–37
22. Kielian M. 2014. Mechanisms of virus membrane fusion proteins. *Annu. Rev. Virol.* 1(1):171–89
23. Connolly SA, Jardetzky TS, Longnecker R. 2021. The structural basis of herpesvirus entry. *Nat. Rev. Microbiol.* 19(2):110–21
24. Vollmer B, Grünewald K. 2020. Herpesvirus membrane fusion - a team effort. *Curr. Opin. Struct. Biol.* 62:112–20
25. Baquero E, Albertini AAV, Gaudin Y. 2015. Recent mechanistic and structural insights on class iii viral fusion glycoproteins. *Curr. Opin. Struct. Biol.* 33:52–60
26. Beilstein F, Abou Hamdan A, Raux H, Belot L, Ouldali M, et al. 2020. Identification of a ph-sensitive switch in vsv-g and a crystal structure of the g pre-fusion state highlight the vsv-g structural transition pathway. *Cell Rep.* 32(7):108042
27. Benton DJ, Nans A, Calder LJ, Turner J, Neu U, et al. 2018. Influenza hemagglutinin membrane anchor. *Proc. Natl. Acad. Sci. USA*. 115(40):10112–17
28. Xiao T, Cai Y, Chen B. 2021. HIV-1 entry and membrane fusion inhibitors. *Viruses*. 13(5):
29. Fontana J, Steven AC. 2013. At low ph, influenza virus matrix protein m1 undergoes a conformational change prior to dissociating from the membrane. *J. Virol.* 87(10):5621–28
30. Winter SL, Golani G, Lolicato F, Vallbracht M, Thiyagarajah K, et al. 2022. The ebola virus vp40 matrix undergoes endosomal disassembly essential for membrane fusion. *BioRxiv*
31. Stauffer S, Feng Y, Nebioglu F, Heilig R, Picotti P, Helenius A. 2014. Stepwise priming by acidic ph and a high k+ concentration is required for efficient uncoating of influenza a virus cores after penetration. *J. Virol.* 88(22):13029–46
32. Moi D, Nishio S, Li X, Valansi C, Langleib M, et al. 2022. Discovery of archaeal

fusexins homologous to eukaryotic hap2/gcs1 gamete fusion proteins. *Nat. Commun.* 13(1):3880
33. Brukman NG, Li X, Podbilewicz B. 2021. Fusexins, hap2/gcs1 and evolution of gamete fusion. *Front. Cell Dev. Biol.* 9:824024
34. Ebel H, Benecke T, Vollmer B. 2022. Stabilisation of viral membrane fusion proteins in prefusion conformation by structure-based design for structure determination and vaccine development. *Viruses*. 14(8):
35. White JM, Delos SE, Brecher M, Schornberg K. 2008. Structures and mechanisms of viral membrane fusion proteins: multiple variations on a common theme. *Crit Rev Biochem Mol Biol*. 43(3):189–219
36. Harrison SC. 2015. Viral membrane fusion. *Virology*. 479–480:498–507
37. White JM, Whittaker GR. 2016. Fusion of enveloped viruses in endosomes. *Traffic*. 17(6):593–614
38. Rey FA, Lok S-M. 2018. Common features of enveloped viruses and implications for immunogen design for next-generation vaccines. *Cell*. 172(6):1319–34
39. Negi G, Sharma A, Dey M, Dhanawat G, Parveen N. 2022. Membrane attachment and fusion of hiv-1, influenza a, and sars-cov-2: resolving the mechanisms with biophysical methods. *Biophys. Rev.*, pp. 1–32
40. Barrett CT, Dutch RE. 2020. Viral membrane fusion and the transmembrane domain. *Viruses*. 12(7):
41. Vaney M-C, Dellarole M, Duquerroy S, Medits I, Tsouchnikas G, et al. 2022. Evolution and activation mechanism of the flavivirus class ii membrane-fusion machinery. *Nat. Commun.* 13(1):3718
42. Ni F, Chen X, Shen J, Wang Q. 2014. Structural insights into the membrane fusion mechanism mediated by influenza virus hemagglutinin. *Biochemistry*. 53(5):846–54
43. Dean TT, Serrão VHB, Lee JE. 2022. Structure of the core postfusion porcine endogenous retrovirus fusion protein. *MBio*, p. e0292021
44. Qiao H, Pelletier SL, Hoffman L, Hacker J, Armstrong RT, White JM. 1998. Specific single or double proline substitutions in the "spring-loaded" coiled-coil region of the influenza hemagglutinin impair or abolish membrane fusion activity. *J. Cell Biol.* 141(6):1335–47
45. Park HE, Gruenke JA, White JM. 2003. Leash in the groove mechanism of membrane fusion. *Nat. Struct. Biol.* 10(12):1048–53
46. Gruenke JA, Armstrong RT, Newcomb WW, Brown JC, White JM. 2002. New insights into the spring-loaded conformational change of influenza virus hemagglutinin. *J. Virol.* 76(9):4456–66
47. Boonstra S, Blijleven JS, Roos WH, Onck PR, van der Giessen E, van Oijen AM. 2018. Hemagglutinin-mediated membrane fusion: a biophysical perspective. *Annu. Rev. Biophys.* 47:153–73
48. Kuhn RJ, Zhang W, Rossmann MG, Pletnev SV, Corver J, et al. 2002. Structure of dengue virus: implications for flavivirus organization, maturation, and fusion. *Cell*. 108(5):717–25
49. Zhang X, Ge P, Yu X, Brannan JM, Bi G, et al. 2013. Cryo-em structure of the mature dengue virus at 3.5-å resolution. *Nat. Struct. Mol. Biol.* 20(1):105–10
50. Klein DE, Choi JL, Harrison SC. 2013. Structure of a dengue virus envelope

protein late-stage fusion intermediate. *J. Virol.* 87(4):2287–93
51. Schmidt AG, Yang PL, Harrison SC. 2010. Peptide inhibitors of dengue-virus entry target a late-stage fusion intermediate. *PLoS Pathog.* 6(4):e1000851
52. Guardado-Calvo P, Rey FA. 2021. The viral class ii membrane fusion machinery: divergent evolution from an ancestral heterodimer. *Viruses*. 13(12):
53. Gibbons DL, Erk I, Reilly B, Navaza J, Kielian M, et al. 2003. Visualization of the target-membrane-inserted fusion protein of semliki forest virus by combined electron microscopy and crystallography. *Cell*. 114(5):573–83
54. Sánchez-San Martín C, Sosa H, Kielian M. 2008. A stable prefusion intermediate of the alphavirus fusion protein reveals critical features of class ii membrane fusion. *Cell Host Microbe*. 4(6):600–608
55. Stiasny K, Bressanelli S, Lepault J, Rey FA, Heinz FX. 2004. Characterization of a membrane-associated trimeric low-ph-induced form of the class ii viral fusion protein e from tick-borne encephalitis virus and its crystallization. *J. Virol.* 78(6):3178–83
56. Vollmer B, Pražák V, Vasishtan D, Jefferys EE, Hernandez-Duran A, et al. 2020. The prefusion structure of herpes simplex virus glycoprotein b. *Sci. Adv.* 6(39):
57. Liu Y, Heim KP, Che Y, Chi X, Qiu X, et al. 2021. Prefusion structure of human cytomegalovirus glycoprotein b and structural basis for membrane fusion. *Sci. Adv.* 7(10):
58. Belot L, Ouldali M, Roche S, Legrand P, Gaudin Y, Albertini AA. 2020. Crystal structure of mokola virus glycoprotein in its post-fusion conformation. *PLoS Pathog.* 16(3):e1008383
59. Abou-Hamdan A, Belot L, Albertini A, Gaudin Y. 2018. Monomeric intermediates formed by vesiculovirus glycoprotein during its low-ph-induced structural transition. *J. Mol. Biol.* 430(12):1685–95
60. Yang F, Lin S, Ye F, Yang J, Qi J, et al. 2020. Structural analysis of rabies virus glycoprotein reveals ph-dependent conformational changes and interactions with a neutralizing antibody. *Cell Host Microbe*. 27(3):441–453.e7
61. Libersou S, Albertini AAV, Ouldali M, Maury V, Maheu C, et al. 2010. Distinct structural rearrangements of the vsv glycoprotein drive membrane fusion. *J. Cell Biol.* 191(1):199–210
62. Vallbracht M, Brun D, Tassinari M, Vaney M-C, Pehau-Arnaudet G, et al. 2018. Structure-function dissection of pseudorabies virus glycoprotein b fusion loops. *J. Virol.* 92(1):
63. Jiao J, Rebane AA, Ma L, Gao Y, Zhang Y. 2015. Kinetically coupled folding of a single hiv-1 glycoprotein 41 complex in viral membrane fusion and inhibition. *Proc. Natl. Acad. Sci. USA*. 112(22):E2855-64
64. Popovic M. 2022. Standard gibbs energy of binding of the gp120 antigen of hiv-1 to the cd4 receptor
65. Yang S-T, Kreutzberger AJB, Kiessling V, Ganser-Pornillos BK, White JM, Tamm LK. 2017. HIV virions sense plasma membrane heterogeneity for cell entry. *Sci. Adv.* 3(6):e1700338
66. Hanne J, Göttfert F, Schimer J, Anders-Össwein M, Konvalinka J, et al. 2016. Stimulated emission depletion nanoscopy reveals time-course of human immunodeficiency virus proteolytic maturation. *ACS Nano*. 10(9):8215–22

67. Chojnacki J, Staudt T, Glass B, Bingen P, Engelhardt J, et al. 2012. Maturation-dependent hiv-1 surface protein redistribution revealed by fluorescence nanoscopy. *Science*. 338(6106):524–28
68. Schwarzer R, Levental I, Gramatica A, Scolari S, Buschmann V, et al. 2014. The cholesterol-binding motif of the hiv-1 glycoprotein gp41 regulates lateral sorting and oligomerization. *Cell Microbiol.* 16(10):1565–81
69. Nieto-Garai JA, Arboleya A, Otaegi S, Chojnacki J, Casas J, et al. 2021. Cholesterol in the viral membrane is a molecular switch governing hiv-1 env clustering. *Adv Sci (Weinh)*. 8(3):2003468
70. Yang S-T, Kiessling V, Simmons JA, White JM, Tamm LK. 2015. HIV gp41-mediated membrane fusion occurs at edges of cholesterol-rich lipid domains. *Nat. Chem. Biol.* 11(6):424–31
71. Yang S-T, Kiessling V, Tamm LK. 2016. Line tension at lipid phase boundaries as driving force for hiv fusion peptide-mediated fusion. *Nat. Commun.* 7:11401
72. Gregory SM, Harada E, Liang B, Delos SE, White JM, Tamm LK. 2011. Structure and function of the complete internal fusion loop from ebolavirus glycoprotein 2. *Proc. Natl. Acad. Sci. USA*. 108(27):11211–16
73. Gregory SM, Larsson P, Nelson EA, Kasson PM, White JM, Tamm LK. 2014. Ebolavirus entry requires a compact hydrophobic fist at the tip of the fusion loop. *J. Virol.* 88(12):6636–49
74. Lee J, Nyenhuis DA, Nelson EA, Cafiso DS, White JM, Tamm LK. 2017. Structure of the ebola virus envelope protein mper/tm domain and its interaction with the fusion loop explains their fusion activity. *Proc. Natl. Acad. Sci. USA*. 114(38):E7987–96
75. Shi W, Cai Y, Zhu H, Peng H, Voyer J, et al. 2022. Cryo-em structure of sars-cov-2 postfusion spike in membrane. *BioRxiv*
